# Genome-wide survey reveals dynamic widespread tissue-specific changes in DNA methylation during development

**DOI:** 10.1186/1471-2164-12-231

**Published:** 2011-05-11

**Authors:** Ping Liang, Fei Song, Srimoyee Ghosh, Evan Morien, Maochun Qin, Saleh Mahmood, Kyoko Fujiwara, Jun Igarashi, Hiroki Nagase, William A Held

**Affiliations:** 1 Department of Biological Sciences, Brock University, St. Catharines, Ontario, Canada; 2 Department of Molecular and Cellular Biology, Roswell Park Cancer Institute, Buffalo, NY, USA; 3 Department of Pharmacology and Therapeutics, Roswell Park Cancer Institute, Buffalo, NY, USA; 4 Department of Cancer Genetics, Roswell Park Cancer Institute, Buffalo, NY, USA; 5 Department of Zoology, North-Eastern Hill University, Umshing Mawkynroh, Shillong, Meghalaya, India; 6 Department of Bioinformatics, University of British Columbia, Vancouver, British Columbia, Canada; 7 Department of Biochemistry, State University of New York at Buffalo, Buffalo, NY, USA; 8 Division of Cancer Genetics, Department of Advanced Medical Science, Nihon University School of Medicine, Tokyo, Japan; 9 Division of Cancer Genetics, Chiba Cancer Center, Research Institute, Chiba, Japan

## Abstract

**Background:**

Changes in DNA methylation in the mammalian genome during development are frequent events and play major roles regulating gene expression and other developmental processes. It is necessary to identify these events so that we may understand how these changes affect normal development and how aberrant changes may impact disease.

**Results:**

In this study Methylated DNA ImmunoPrecipitation (MeDIP) was used in conjunction with a NimbleGen promoter plus CpG island (CpGi) array to identify Tissue and Developmental Stage specific Differentially Methylated DNA Regions (T-DMRs and DS-DMRs) on a genome-wide basis. Four tissues (brain, heart, liver, and testis) from C57BL/6J mice were analyzed at three developmental stages (15 day embryo, E15; new born, NB; 12 week adult, AD). Almost 5,000 adult T-DMRs and 10,000 DS-DMRs were identified. Surprisingly, almost all DS-DMRs were tissue specific (i.e. methylated in at least one tissue and unmethylated in one or more tissues). In addition our results indicate that many DS-DMRs are methylated at early development stages (E15 and NB) but are unmethylated in adult. There is a very strong bias for testis specific methylation in non-CpGi promoter regions (94%). Although the majority of T-DMRs and DS-DMRs tended to be in non-CpGi promoter regions, a relatively large number were also located in CpGi in promoter, intragenic and intergenic regions (>15% of the 15,979 CpGi on the array).

**Conclusions:**

Our data suggests the vast majority of unique sequence DNA methylation has tissue specificity, that demethylation has a prominent role in tissue differentiation, and that DNA methylation has regulatory roles in alternative promoter selection and in non-promoter regions. Overall, our studies indicate changes in DNA methylation during development are a dynamic, widespread, and tissue-specific process involving both DNA methylation and demethylation.

## Background

DNA methylation in mammals occurs predominantly at CpG dinucleotides [1]. Early studies indicated that in normal cells, most CpGs in repeats, retroviral sequences, and within the coding region of genes are methylated, whereas most CpG island (CpGi) regions are maintained in an unmethylated state [2,3]. After fertilization there is a period of genome-wide demethylation followed by periods of remethylation in somatic cells after implantation. Demethylation and reprogramming is also necessary in the germ cells to reset gamete-specific imprinting [4-7]. There is a long history and generally inverse correlation between gene promoter DNA methylation and gene expression [8-12]. Early studies proposed that regulated methylation and demethylation has a role regulating gene expression during development [10,13].

The critical function of DNA methylation during development is also apparent from the consequence of targeted knockouts of DNA methyltransferase genes. The knockout of *Dnmt1*, which has a strong preference for hemimethylated DNA and is thus considered to be a maintenance DNA methyltransferase, is lethal during development [14]. *Dnmt3a *and *Dnmt3b *are required for *de novo *methylation during development [15]. The persistent expression of both *Dnmt *3a and *Dnmt *3b after gastrulation and implantation suggests that *de novo *methylation occurs during later stages of development.

Restriction Landmark Genomic Scanning (RLGS) detected differences in DNA methylation between tissues more than 15 years ago [16-19]. These studies, as well as more recent reports using a variety of methods, have markedly altered our concept of differentially methylated regions (DMRs) in the mammalian genome and the progression of DNA methylation changes during development [19-36]. Tissue Specific Differentially Methylated Regions (T-DMRs) include both CpGi and non-CpGi promoter regions as well as intragenic and intergenic regions [19,32,37,38]. Analysis of T-DMRs using a CpGi array indicated that T-DMRs in CpGi regions are associated with developmental gene loci and many are located in non-promoter regions [23]. CpGi amplification in conjunction with microarrays, indicated approximately 4% of dense CpGi promoters are methylated in normal peripheral blood [31]. A number of studies have consistently observed that weak or non-CpGi promoters are major targets for tissue specific DNA methylation [12,28,30,33,39].

Analysis of DNA methylation changes during development *in vitro *and *in vivo *are more limited but indicate that DNA methylation is a dynamic process involving both methylation and demethylation [20,26,32]. *In vivo *analysis of methylation changes within a tissue at different developmental stages is complicated by changes in cell populations [32]. *In vitro *analysis of DNA methylation during differentiation of ES cells or progenitor cells is complicated by potential aberrant methylation due to growth of cells in tissue culture [20,26,40].

Our previous results identified a limited number of T-DMRs using RLGS, suggesting that methylation changes during development are dynamic and involve both methylation and demethylation [19,32]. The results reported here investigate this with a more comprehensive set of T-DMRs and Developmental Stage specific Differentially Methylated DNA Regions (DS-DMRs) identified through Methylated DNA ImmunoPrecipitation (MeDIP) [20,32].

## Results

### MeDIP methylation analysis

The MeDIP methylation analysis developed by Weber et al [33] was utilized to identify genomic sites of differential DNA methylation in selected 12 wk adult C57BL/6J mouse tissues (T-DMRs) and in tissues at different developmental stages (DS-DMRs: 15 day embryo, E15; new born, NB; and 12 wk adult, AD). Methylated DNA was immunoprecipitated with antibody to 5-methyl-cytidine, the input and immunoprecipitated (IP) DNA were differentially labeled and hybridized to a NimbleGen promoter plus CpGi array according to a protocol provided by NimbleGen. The NimbleGen Array covers all UCSC-annotated CpGi (15,979) as well as promoters for all RefSeq genes (19,530). Repetitive regions are excluded from the Array. Non-CpGi intragenic and intergenic regions are not covered by the array except in some limited regions in which the array is essentially a tiling array (1.9 Mb on portions of chromosomes 6, 7, and 17).

### Assessment of the MeDIP data quality and validation of MeDIP methylation peaks

**Figure 1:**
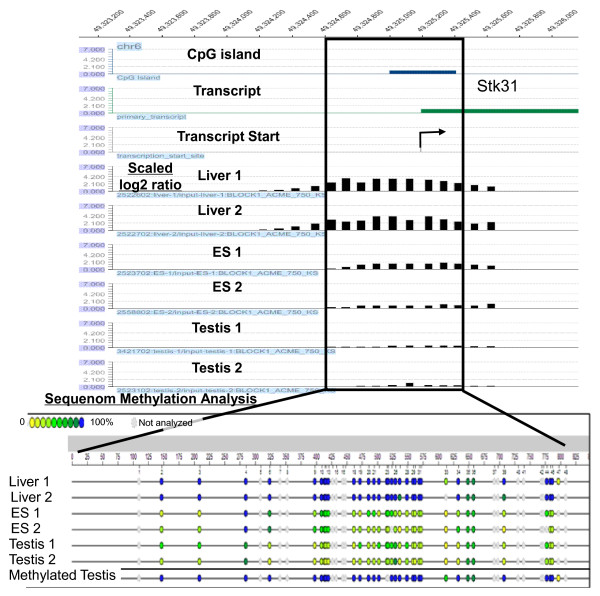
**Comparison of methylation data between MeDIP/NimbleGen Promoter + CpGi Array and Sequenom MassArray: Stk31 CpGi promoter region**. The *Stk31 *gene region with a CpGi promoter region that is methylated in the liver, but not in ES or testis cells. The box indicates the position of the methylation peak in the liver that overlaps with the CpGi promoter region analyzed by Sequenom. In the epigram of Sequenom methylation analysis shown at the bottom panel, the blue circles indicate 100% methylation, green circles indicate around 50%methylation and yellow circles indicate 0% methylation. The results from biological duplicate samples are shown. The bottom lane (Sequenom Methylation Analysis) shows the results of analysis of testis DNA after *in vitro *methylation using M. Sss1 methylase (New England Biolabs).

A number of methods were used to validate the quality of the MeDIP methylation data. These include scatter plots of log 2 ratios between IP DNA and input DNA, analysis of Pearson Coefficients between biological replicate samples, comparison of MeDIP methylation with previously identified RLGS T-DMRs, MeDIP analysis of other known methylation sites, such as imprinted genes, and analysis of both selected and randomly chosen MeDIP methylated regions by Sequenom MassARRAY quantitative methylation analysis of bisulfite treated DNA. An example of a selected region analyzed by Sequenom MassARRAY is shown in Figure 1. The results indicate a close similarity between MeDIP and Sequenom MassARRAY methylation analysis. Additional details of Sequenom MassARRAY methylation analysis and other validation methods are presented in the Methods section and Additional files.

### Identification of genomic sites of DNA methylation in adult tissues (brain, heart, liver, and testis)

The IP and input DNA from 12 wk adult C57BL/6J mouse tissues (brain, heart, liver, and testis) were hybridized to a NimbleGen Promoter plus CpGi array. T-DMRs are genomic regions that are methylated in one or more of the four adult tissues, but unmethylated in at least one tissue. For the identification of T-DMRs, the log2 ratios of all probes for each sample were normalized and used to identify methylation peaks as described in the methods. To facilitate the analysis of MeDIP data, an *ad hoc *bioinformatics tool was developed to process the methylation peak lists for individual samples and identify genomic regions that are 1) commonly methylated in all tissues; 2) methylated in a single tissue; and 3) methylated in some but not all tissues. Only those methylation peak regions that were present in both biological replicate samples and were absent from at least one other tissue were designated as T-DMRs. Thus, the frequency of T-DMRs may be underestimated. T-DMR locations were designated as 1) CpGi Promoter; 2) Intragenic CpGi; 3) Intergenic CpGi; and 4) Non-CpGi promoter (see details in Methods).

Summaries of the number and distributions of the adult T-DMRs are shown in Table 1. Overall, there are almost 5,000 adult T-DMRs that are present in one or more of the examined adult tissues but are absent in one or more tissues, whereas there are only 460 methylated regions that are common to all four tissues. There are a relatively high number of T-DMRs unique to testis (1,294) and a relatively low number unique to brain (285). Most of the T-DMRs (69.3%) are located within non-CpGi promoter regions, but 30.7% are within CpGi regions, among which 13.7% are within CpGi promoter regions and 17% are in non-promoter CpGi regions (intragenic and intergenic CpGi regions). Within the different tissues there are some notable differences. For example, approximately 94% of the testis-specific T-DMRs are in non-CpGi promoter regions, whereas each of the three somatic tissues is no more than 60%. T-DMRs that are present in brain, heart, and liver, but not testis, would include somatic methylation sites. The majority of these (B+H+L) are in CpGi regions (64.3%).

**Table 1:** Adult T-DMRs

Tissue	Total	CpGi Prom	%	Non-CpGi Prom	%	Intra-genic CpGi	%	Inter-genic CpGi	%
**Common**	460	18	3.9	230	50.0	163	35.4	49	10.7
**Brain unique**	285	68	23.9	173	60.7	23	8.1	21	7.4
**Heart unique**	822	223	27.1	428	52.1	115	14.0	56	6.8
**Liver unique**	711	155	21.8	428	60.2	66	9.3	62	8.7
**Testis unique**	1294	44	3.4	1215	93.9	20	1.5	15	1.2
**Total T-unique**	3112	490	15.7	2244	72.1	224	7.2	154	4.9
**B + H**	482	69	14.3	282	58.5	89	18.5	42	8.7
**B + L**	12	2	16.7	7	58.3	2	16.7	1	8.3
**B + T**	26	1	3.8	25	96.2	0	0.0	0	0.0
**H + L**	49	7	14.3	24	49.0	13	26.5	5	10.2
**H + T**	47	0	0.0	45	95.7	2	4.3	0	0.0
**L + T**	305	3	1.0	268	87.9	27	8.9	7	2.3
**B + H + L**	414	65	15.7	148	35.7	127	30.7	74	17.9
**B + H + T**	175	3	1.7	151	86.3	19	10.9	2	1.1
**B + L + T**	18	0	0.0	15	83.3	2	11.1	1	5.6
**H + L + T**	46	4	8.7	37	80.4	4	8.7	1	2.2
**Total**									
**multi-tissues**	1574	154	9.8	1002	63.7	285	18.1	133	8.4
**Total T-DMRs**	4686	644	13.7	3246	69.3	509	10.9	287	6.1

### The number and distribution of T-DMRs in ES cells

ES cells are derived from the inner cell mass of the early blastocyst that gives rise to the embryo. Following fertilization, the embryonic cells undergo a period of demethylation and make up/form the inner cell mass. Remethylation of somatic tissues is thought to resume following implantation [7]. Thus, we compared the methylation status of T-DMRs in E15 tissues with ES cells (Additional file 1). Overall, there are fewer T-DMRs (2750) from the comparison of ES cell and E15 tissues than T-DMRs in adult tissues (4686, Table 1). We found more unique methylation peaks in ES cells (981) than in E15 tissues of brain (310), heart (131), liver (764), or testis (230). This indicates that there are many sites that are methylated in ES cells but are unmethylated in E15 tissues with most (85.5%) located in non-CpGi promoter regions.

### T-DMRs in regions outside CpGi regions and promoters

The NimbleGen promoter + CpGi array contains a tiling array that covers about 1.9 Mb of the mouse genome. This includes the *Hoxa *gene cluster on chromosome 6 (423 Kb), the *Igf2r *imprinted region on chromosome 17 (88 Kb) and imprinted regions on chromosome 7 (1,390 Kb). In these regions it is possible to identify tissue specific methylation peaks that are not associated with CpGi regions or promoters. For example, in the KvDMR region on chromosome 7 (Additional file 2), there are non-CpGi and non-promoter methylation peaks in testis and brain that are not present in liver in addition to T-DMRs in CpGi regions. The results shown in Additional file 3a indicate that there is similar distribution of T-DMRs in the tiling regions in CpGi and non-CpGi promoter regions as in the rest of the array-covered genome. However, there are additional methylation peaks within the tiling regions (Additional file 3b) in non-CpGi intragenic regions (15.7%) and in non-CpGi intergenic regions (12.9%). These results indicate that almost 30% of the T-DMRs may be missed by restricting analysis to CpGi and promoter regions.

### Developmental Stage-Specific Differentially Methylated Regions (DS-DMRs)

**Figure 2:**
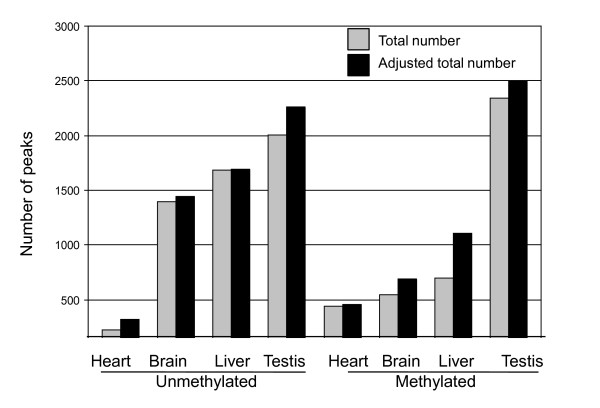
**DS-DMRs: Number of peaks that are Unmethylated or Methylated in adult Tissues**. Total numbers of unmethylated peaks and methylated peaks are shown in adult heart, brain, liver and testis. The grey bar indicates the actual total number of unmethylated and methylated peaks in each tissue. The black bar indicates total number of unmethylated and methylated peaks in each tissue after adjustment for differences in the Pearson Coefficient.

We used MeDIP to determine the number and location of methylated sites at the different developmental stages within each tissue **(**Table 2**)**. DS-DMRs are genomic regions that are methylated at one or more developmental stage of a tissue, but unmethylated in at least one stage. It should be noted that DS-DMRs are selected as stage-specific differentially methylated regions within a tissue without regard to presence or absence in another tissue. This analysis provides a basis for determining changes in developmental stage specific methylation that occur within a tissue. These changes may reflect active or passive changes in DNA methylation as well as changes in the proportion of specific cell populations. The Pearson Coefficients between the duplicate biological replicates varied somewhat from sample to sample (Additional file 4). Therefore the total number of methylation peaks would be somewhat underestimated in samples with low relative to those with high Pearson Coefficients. We adjusted the total number of methylation peaks as if the Pearson Coefficients for all stages were the same as the best one in that tissue (see data labeled "Adjusted" in Table 2). Although the total number of peaks increases for the samples with lower Coefficients, the overall trends are comparable (Table 2 and Figure 2), indicating that our underestimation of methylation peaks does not have a significant impact on the distribution of methylation peaks into different genomic regions.

**Table 2:** The number of DS-DMRs in different tissues

	Total	Adjusted^#^	%^1^	CpGi-Prom	%^2^	non-CpGi-Prom	%^2^	Intra-genic CpGi	%^2^	Inter-genic CpGi	%^2^
**Brain:**											

**Common**	1903	NA	ND	182	9.6	1018	53.5	493	25.9	210	11.0
**E15_uniq**	533	533	27.5	105	19.7	359	67.4	35	6.6	34	6.4
**E15+NB**	490	509	25.3	66	13.5	368	75.1	29	5.9	27	5.5
**NB_uniq**	370	400	19.1	55	14.9	248	67.0	42	11.4	25	6.8
**AD_uniq**	408	532	21.1	94	23.0	233	57.1	52	12.7	29	7.1
**NB+AD**	70	82	3.6	5	7.1	40	57.1	16	22.9	9	12.9
**E15+AD**	65	77	3.4	11	16.9	46	70.8	7	10.8	1	1.5
Total*	**1936**	**2132**	**100.0**	**336**	**15.2**	**1294**	**62.6**	**181**	**13.9**	**125**	**8.4**
UnMe-AD**	1393	1442	72.0	226	16.2	975	70.0	106	7.6	86	6.2
Me-AD***	543	691	28.0	110	20.3	319	58.8	75	13.8	39	7.2

**Heart:**											

**Common**	1844	NA	ND	218	11.8	946	51.3	491	26.6	189	10.2
**E15_uniq**	46	81	6.9	27	58.7	8	17.4	2	4.3	9	19.6
**E15+NB**	87	125	13.0	17	19.5	61	70.1	4	4.6	5	5.7
**NB_uniq**	93	112	13.9	12	12.9	64	68.8	7	7.5	10	10.8
**AD_uniq**	172	172	25.6	45	26.2	83	48.3	33	19.2	11	6.4
**NB+AD**	246	253	36.7	44	17.9	132	53.7	54	22.0	16	6.5
**E15+AD**	27	34	4.0	6	22.2	15	55.6	5	18.5	1	3.7
Total*	**671**	**777**	**100.0**	**151**	**22.4**	**363**	**54.1**	**105**	**15.6**	**52**	**7.7**
UnMe-AD**	226	318	33.7	56	24.8	133	58.8	13	5.8	24	10.6
Me-AD***	445	459	66.3	95	21.3	230	51.7	92	20.7	28	6.3

**Liver:**											

**Common**	1497	NA	ND	141	9.4	726	48.5	425	28.4	205	13.7
**E15_uniq**	117	119	4.9	25	21.4	74	63.2	10	8.5	8	6.8
**E15+NB**	1419	1431	60.0	198	14.0	1034	72.9	125	8.8	62	4.4
**NB_uniq**	147	147	6.2	24	16.3	109	74.1	8	5.4	6	4.1
**AD_uniq**	468	813	19.7	70	15.0	322	68.8	45	9.6	31	6.6
**NB+AD**	36	46	1.5	2	5.6	30	83.3	3	8.3	1	2.8
**E15+AD**	194	249	8.1	13	6.7	130	67.0	31	16.0	20	10.3
Total*	**2381**	**2804**	**100.0**	**332**	**13.9**	**1699**	**71.4**	**222**	**9.3**	**128**	**5.4**
UnMe-AD**	1683	1696	70.7	247	14.7	1217	72.3	143	8.5	76	4.5
Me AD***	698	1107	29.3	85	12.	482	69.1	79	11.3	52	7.4
**Testis:**											
**Common**	570	NA	ND	20	3.5	363	63.7	148	26.0	39	6.8
**E15_uniq**	272	380	6.3	105	38.6	101	37.1	33	12.1	33	12.1
**E15+NB**	939	1094	22.0	193	20.6	480	51.1	157	16.7	109	11.6
**NB_uniq**	798	789	18.4	231	28.9	391	49.0	86	10.8	90	11.3
**AD_uniq**	2214	2336	50.9	71	3.2	1908	86.2	165	7.5	70	3.2
**NB+AD**	63	65	1.4	3	4.8	48	76.2	9	14.3	3	4.8
**E15+AD**	62	91	1.4	5	8.1	38	61.3	14	22.6	5	8.1
**Total**	**4348**	**4755**	**100.0**	**608**	**14.0**	**2966**	**68.2**	**464**	**10.7**	**310**	**7.1**
UnMe-AD**	2009	2263	40.8	529	26.3	972	48.4	276	13.7	232	11.5
Me-AD***	2339	2493	47.6	79	3.4	1994	85.3	188	8.0	78	3.3

**Grand Total:**											

**Common**	**5814**	**NA**	**ND**	**561**	**9.6**	**3053**	**52.5**	**1557**	**26.8**	**643**	**11.1**
**DS-DMRs**	**9336**	**10469**	**100.0**	**1427**	**15.3**	**6322**	**67.7**	**972**	**10.4**	**615**	**6.6**
UnMe-AD**	**5311**	**5719**	**56.9**	**1058**	**19.9**	**3297**	**62.1**	**538**	**10.1**	**418**	**7.9**
Me-AD***	**4025**	**4750**	**43.1**	**369**	**9.2**	**3025**	**75.2**	**434**	**10.8**	**197**	**4.9**

**%^1^**: percentage is based on the row total vs. total DMRs; %^2^: percentage is based on the total of DMRs in the row; Total*: Stage specific; UnMe-AD**: unmethylated in AD but methylated in E15 and/or NB; Me-AD***: methylated in Adult but unmethylated in E15 and/or NB; Adjusted#: number of DMRs adjusted based on the adjusted coefficiency between the duplicated samples.

There were considerably more methylation sites that were common within a tissue at the different developmental stages than between different adult tissues (460 common sites, Table 1). Brain, heart, and liver each had 1,903, 1,844 and 1,497 common methylation sites, respectively, whereas testis had 570 (Table 2). Similar to the common sites for the adult tissues, about 50% of developmental stage common sites are in CpGi regions (CpGi promoter + intragenic and intergenic CpGi) and about 50% are in non-CpGi promoter regions (Table 1 and 2**)**. Somewhat surprising is the relatively high number of sites in liver (and brain and testis, to some degree) that are methylated in E15 + NB but unmethylated in adult (1,419). Together, in somatic tissues (heart, brain, and liver), there were almost 5,000 sites of methylation that exhibit stage specific differences. Testis alone had 4,348 sites of stage specific methylated regions, most of which were methylated in adult and localized to non-CpGi promoters. Again, as for the adult tissues, the most frequent location for differential methylation was within non-CpGi promoter regions (54.1% to 71.4%), followed by CpGi promoter regions (13.9 to 22.4%), intragenic CpGi regions (9.3 to 15.6%), and intergenic CpGi regions (5.4 to 8.4%). In contrast to other tissues, the location of testis stage specific DS-DMRs appear to shift from a high proportion of CpGi location at early developmental stages (E15, 62.9% and NB, 51.0%) to a high proportion of non-CpGi promoter location in adult (86.2%).

### Many DS-DMRs are unmethylated in adult

We grouped the DS-DMRs within a tissue according to whether they were unmethylated in adult (UnMe-AD) but methylated at earlier developmental stages (E15, E15+NB, NB). These would represent sites of demethylation during development. Conversely sites that were methylated in adult (E15+AD, NB+AD, AD) but unmethylated at earlier developmental stages would be sites that were methylated during development. Surprisingly, there were more sites that became unmethylated (5,311) than became methylated (4,025) during development (Figure 2 and Table 2). Adjustment for differences in the Pearson Coefficient (Figure 2) does not significantly alter this general conclusion. Among the different tissues, there seem to be some significant differences with regard to the ratio of DMRs subjected to methylation and demethylation. In testis, there are an almost equal number of sites that become unmethylated (2,009) or methylated (2,339) in adult. However, in brain and liver there are a much larger number of sites undergoing demethylation in adult than that of methylation whereas in heart, there are a smaller number of sites undergoing demethylation than methylation. These results indicate dynamic changes in DNA methylation during development and that epigenetic changes resulting in unmethylated DS-DMRs are a major feature of differentiation into adult tissues.

### Overlap between DS-DMRs in different tissues

The DS-DMRs indicate differential stage-specific sites of methylation within a tissue without regard to whether it is tissue specific. To determine how many of the DS-DMRs are tissue specific, we determined the extent of overlapping DS-DMR (i.e., methylation) between tissues (Table 3). The results indicate for all four tissues together, the majority (7,857 or 80.3%) of the 9,784 DS-DMRs are unique to a single tissue whereas the remaining 1,927 DS-DMRs are present in two or more but not all tissues, and thus are also tissue-specific. Together, it indicates that all of the 9,784 DS-DMRs are also T-DMRs. If testis is excluded, there are 4,232 out of 5,519 DS-DMRs that are unique to a single tissue and 613 peaks being present from one or more but not all tissues (Additional file 5a). In this case, we did find a small number of peaks (21) that are common to all three tissues, suggesting that the 3 somatic tissues share more commonality among each other than with testis. We also examined the overlap for the 5,761 DMRs that are common to all stages in a tissue (Additional file 5b). The majority of these DMRs are also tissue-specific (5,487/5,761 or 95.2%). However, compared to DS-DMRs, the percentage of these DMRs being tissue-unique is much lower, approximately 15% for each of the somatic tissues and only 1.9% for testis. Only a small proportion (274 or 4.8%) are common to all developmental stages and all tissues. Overall, since all of the 9,784 DS-DMRs and 5,487 of the 5,761 DMRs common to all developmental stages in a tissue are also T-DMRs, we identified a total of 15,271 T-DMRs.

**Table 3:** Overlap among DS-DMRs from different tissues

Tissue	all DS-DMRs	Tissue-Unique	Multiple tissues#	T-DMR%
**Brain**	1973	1065	908	100
**Heart**	867	533	334	100
**Liver**	3570	2160	1410	100
**Testis**	5469	4099	1370	100
**Total**	11879*/9784**	7857	4022*/1927**	

#: peak present in 2 or more but not all tissues; *: contains redundancy due to overlap; The numbers are larger than those in Table 2 due to certain peak splitting; **: a non-redundant (unique) list of all sites from the four tissues.

### Tissue-Specific differences in DNA methylation within the Hoxa gene cluster

**Figure 3:**
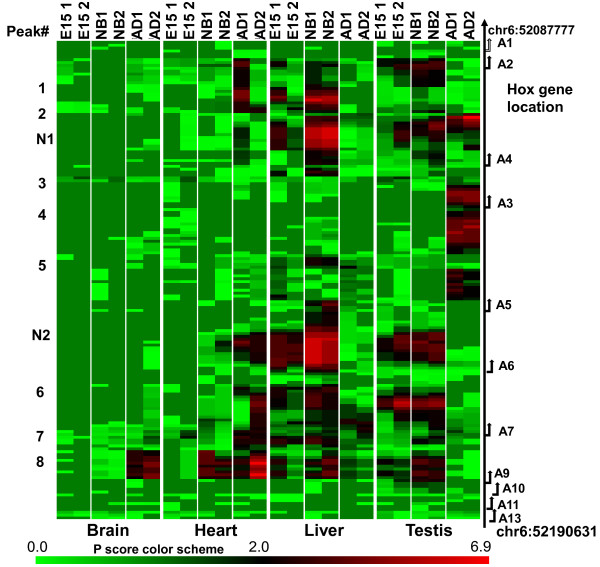
**DS-DMRs: Heat Map of methyation signal in the *Hoxa *gene region during development in different tissues**. A heat map was constructed based on the NimbleGen P scores of probes, in the *Hoxa *gene cluster region. Probes included in the heat map either have a P score ≥2 for both of the duplicated samples or at least one tissue/developmental stage or fall into the promoter region of a *Hoxa *gene. Each row of the color tiles in the heat map represents a NimbleGen probe, while each column represents a sample. The tissue name is labeled at the bottom, while the developmental stage is indicated at the top. The labeled arrows at the right side indicate the position of the TSS and direction of the *Hoxa *gene transcription. The T-DMRs and DS-DMRs methylation peaks corresponding to those in Additional file 14b are indicated by the numbers on the left side.

Homeobox genes have important roles in regulating development [41,42]. One of the tiled regions available on the NimbleGen array includes the Hoxa gene cluster on chromosome 6. Additional file 6 shows the scaled log2 ratios from the NimbleGen SignalMap view of an approximately 100 Kb region that includes the *Hoxa *gene cluster. There are 8 adult T-DMRs in this region that were present in both biological replicate DNA samples of the involved tissue(s). Even though this region is CpGi rich, none of the peaks were within CpGi regions and only one was localized in a promoter region (Additional file 7; peak 7, *Hoxa7*). The other methylation peaks were within introns (peaks 2, 3, and 4) or associated with the 3' end (exon) of the gene (peaks 1, 5, 6, and 8). Four of the peaks (peaks 2-5) were testis specific, one (peak 6) was heart specific, one (peak 7) was present in heart and liver, and one (peak 8) was in heart and brain. All sequences corresponding to these peaks were highly conserved, suggesting a regulatory function. Analysis of methylation differences within the *Hoxa *gene cluster during development (Figure 3) indicates that two additional DS-DMRs not seen as T-DMRs for adult tissues were observed when samples from all three developmental stages were examined (Additional file 6; Additional file 7). One of these is specific to NB liver (peak N1) and the other is a large region (peak N2) methylated in E15 and NB of liver and testis and in AD heart, and demethylated in adult liver and testis. Overall, our results indicate extensive tissue and developmental stage specific methylation in the *Hoxa *gene cluster that is not associated with promoter or CpGi regions.

### DNA methylation within the Protocadherin gene cluster

Protocadherins are a large gene family involved in cell adhesion and signaling, particularly during neural development. There are three large clusters totaling more than 50 genes (alpha, beta and gamma) that span about 1 MB on Chromosome 18 in the mouse. Additional file 8 shows the MeDIP methylation profile from a portion of the *Pcdha *and *Pchdhg *gene clusters. Many of the alternative promoters are associated with CpG islands. Most of the alternative promoters are completely unmethylated in the adult testis, but some are differentially methylated in the liver (*Pcdhga3 *and *Pcdhgb2*, peaks 8 and 11; Additional file 8 and Additional file 9). Note that the promoter for *Pcdhga6 *(peak 13) is methylated in the testis but unmethylated in liver, brain, and heart. These results indicate that alternative promoters within the Protocadherin gene cluster are targets for DNA methylation.

### Relationship between T-DMRs/DS-DMRs and gene expression

To test whether the T-DMRs and DS-DMRs have any relationship with and the expression of their associated genes, we identified (from NCBI GEO database) and analyzed two published expression data sets, which have the best match to the samples we used in this study with regard to animal strain, tissue type and developmental stage.

For T-DMRs, we used GSE9954 [43] designed for the analysis of 22 different tissues from adult C57BL/6 mice, and we performed a small-scale manual examination, as well as computational analysis for all T-DMRs. Manual examination of individual genes revealed a variety of situations, with no obvious consistent pattern, likely due to reasons discussed later. Nevertheless, the data from the analysis of all T-DMRs seems to reveal two general trends: 1) Genes containing T-DMRs in non-CpGi promoters tend to have lower expression in the tissue to which the T-DMRs are unique than in other three tissues (Additional file 10 panel A-C); 2) Within the same tissue, genes containing T-DMRs in non-CpGi promoters have lower expression than those containing T-DMRs in either in CpGi promoters or in intragenic CpGi and those containing no T-DMRs (Additional file 10 panel D). The first trend is well shown in brain and liver, but less consistent in heart and brain, while the second trend is consistently observed in all four tissues. T-DMRs in CpGi promoters do not seem to associate with a significantly lower expression except for those from liver, while T-DMRs in intra-genic CpGi do not seem to have a significant impact on gene expression either way in any tissue examined. The trend for DS-DMRs, based on analysis of a liver development expression data set (GSE13149 [44]), seems to be less clear except for adult unique DS-DMR, which are mostly T-DMRs as described earlier and show a similar pattern as T-DMRs (data now shown), suggesting that there might be some differences between T-DMRs and DS-DMRs in the ways they impact gene expression.

## Discussion

### Total number of T-DMRs and DS-DMRs

MeDIP methylation analysis in conjunction with NimbleGen promoter + CpGi microarrays were used to identify adult tissue specific differentially methylated regions (T-DMRs) and developmental stage specific DMRs (DS-DMRs). We used relatively conservative criteria to identify methylation peaks requiring both independently derived biological replicates to contain the same or overlapping methylation peaks. Other investigators have indicated that MeDIP may miss differentially methylated regions in regions of low CpG density [45]. Thus we may underestimate the total number of T-DMRs and DS-DMRs to some extent. Nevertheless, our study identified almost 5,000 adult T-DMRs and 10,000 DS-DMRs that represent a total of over 15,000 T-DMRs in four adult tissues (brain, heart, liver, and testis; Tables 1 and 2). Since tissues are made up of many cell types and it is likely that there are cell type specific epigenetic differences, the methylation peaks we identified probably correspond to the major cell types in the tissue, while many methylation peaks corresponding to minor cell types would be missed. Considering that we only sampled 4 tissues and 3 developmental stages (E15, NB, AD), and that the analysis does not include methylation peaks in regions outside promoter and CpGi regions (except in tiling regions), it is quite likely that there are many additional T-DMRs and DS-DMRs that were not identified in this study. These results demonstrate that there are numerous DNA methylation differences between adult tissues (T-DMRs) and within the same tissue at different stages of development (DS-DMRs), indicating that alterations in DNA methylation are a major feature of development.

### Genomic locations of T-DMRs and DS-DMRs

Overall most T-DMRs and DS-DMRs (about 70%) are located within non-CpGi promoter regions (Table 1 and 2), which is consistent with results of other studies [12,28,30,33,39]. However, we also found that about 30% of T-DMRs (1,440/4,686) and DS-DMRs (3,014/9,336) are located in CpGi regions (i.e. the sum of all CpGi-associated DMRs vs. the total number of DMRs; Table 1 and 2). This corresponds to more than 15% of the 15,979 CpGi regions on the NimbleGen array and is a relatively large number considering the limited number of tissues and developmental stages surveyed and other factors cited above. In fact, among the 15,979 CpGi regions annotated in UCSC mm8 freeze, a total of 5,523 (34.6%) were partially or entirely methylated in one or more of the 24 samples analyzed in this study (4 tissues each with duplicated samples for 3 developmental stages; data not shown). Thus, tissue- and developmental stage-specific CpGi methylation may be a very common event during development.

Analysis of methylation in the few tiling regions available on the NimbleGen array made it possible to determine whether there was significant tissue specific methylation outside of promoter and CpG island regions. Our results indicate that as much as 30% of the T-DMRs are missed by restricting analysis to promoter and CpGi regions (Additional file 3b).

The location of methylation peaks differs according to tissue distribution. About 50% of the adult common methylation sites are within CpGi regions whereas only 30% of the T-DMRs are located within CpGi regions (Table 1). Similarly, about 48% of the methylation peaks that are common among all developmental stages within a tissue are located in CpGi regions (Table 2**)**. The significance of this difference between common methylation and differential methylation is not presently clear. Possibly this reflects a difference between transient changes in methylation that are tissue specific and more stable changes in methylation that are common to tissues.

The distribution of the location of T-DMRs and DS-DMRs in testis is dramatically different from somatic tissues. Almost 94% of the adult testis T-DMRs are associated with non-CpGi promoter regions (Table 1 and 2). About 68% of the testis DS-DMRs are associated with non-CpGi promoter regions, which represents an average of E15, NB, and AD developmental stages. Unlike somatic tissues there appears to be a clear pattern shift in the distribution of testis DS-DMRs during development. In E15 testis only about 37% of the DS-DMRs are located in non-CpGi promoters whereas the percentage increases in NB (49%) and AD (86%). This may reflect post-natal onset of meiosis and other developmental changes. This general bias of DMRs towards non-CpGi promoter vs. CpGi promoter is significant since the majority (10,915 or 68.5%) of 19,528 promoters (all RefSeqs promoters included in the array) are CpGi promoters (based on our criteria region of 1kb flanking each side of transcription start site).

T-DMRs correspond to sites of methylation that vary between tissues, whereas DS-DMRs correspond to sites of methylation within a single tissue that differ according to developmental stage. We determined the extent of overlap between DS-DMRs from different tissues by combining all DS-DMRs for the same tissue and excluding common methylation sites within a tissue (Table 3 and Additional file 5a and 5b). We reasoned that the degree of overlap of DS-DMRs among tissues would shed light on the level of tissue specificity of DNA methylation during development. We found a very low level of overlap among different tissues (1,927/9,784 or 13.2%), indicating that most DS-DMRs are tissue-unique and tissue-specific (Table 3). Even for DMRs that are shared among all three developmental stages in a tissue (Additional file 5b), almost 50% are tissue unique and almost all are tissue specific (95.2%). In addition, a large fraction of the tissue specific DS-DMRs were unique to a single tissue (7,857/9,784 or 80%). However, we would expect this percentage to decrease as more tissues are added to the analysis. These results suggest that almost all DNA methylation in non-repetitive regions is tissue specific. We have previously shown, using RLGS, that methylation of some genomic regions containing repetitive sequences is also tissue-specific [32].

Recent studies [22,25] identified 16,379 T-DMRs in four human adult tissues (brain, liver, spleen, and colon) using a method termed "Comprehensive High-throughput Arrays for Relative Methylation" or CHARM. Although the methods, species and tissues used for this analysis were different from those presented here, the total number of T-DMRs is surprisingly similar (15,271 vs. 16,379), emphasizing extensive tissue specific DNA differences in DNA methylation. However, the CHARM analysis of human tissues found that 76% of the T-DMRs were located within 2 kb of CpGi regions that were denoted CpGi shores. The array design used in our study limits analysis to promoter, CpGi regions, and very limited tiled genomic regions and would appear to exclude most CpGi shores. However, we reason that many CpGi shores may be within the promoter regions in our studies. For both the promoter and tiling regions, we observed a slightly higher density of DS-DMRs in CpGi shores than in CpGi regions (data not shown). Since at the genome scale, the CpGi shore region is much larger (~6X) than the CpGi region, we can expect to have a larger number of DS-DMRs in the CpGi shores than in the CpGi, supporting the conclusion of Irizarry et al [25] in principle that there are more T-DMRs in CpGi shores.

### Gene ontology of T-DMRs and DS-DMRs

Gene ontology (GO) analysis was performed to identify any common theme among the identified T-DMRs. In this analysis, we divided all DMRs associated with genes into three groups (non-CpGi promoter, CpGi promoter, and intragenic CpGi), since as noted by others [37,38], methylation in different gene locations may impact the gene expression regulation differently. We found more or less similar enrichment categories for different tissues, especially among somatic tissues. For both T-DMRs and DS-DMRs, the most consistent GO enrichment pattern is seen among those in non-CpGi promoters (Additional file 11 and Additional file 12) with enrichment for "membrane proteins, G-proteins, olfactory proteins" among UnMe-AD (DS-DMRs) and "extracellular space/region" for both T-DMRs and DS-DMRS (Me-AD). Apparently, these enrichment patterns reflect a pattern that is observed for all genes with non-CpGi promoters (data not shown). Additional GO enrichment was observed for somatic DS-DMRs in intragenic CpGi regions on "regulation of biological and cellular processes, ion-binding and transport". This data supports the notion that intra-genic CpGi methylation tends to promote the expression of the involved gene [46,47], and may participate in gene regulation during differentiation and development.

### "Demethylation" of previously methylated sites (DS-DMRs) is a common feature of tissue differentiation

Quite surprisingly, our studies indicate that many DS-DMRs that are methylated at early stages of development (E15 and NB) are unmethylated in adult tissues (Table 2 and Figure 2). This is particularly evident in brain and liver where there are almost 3 times as many DS-DMRs that become "unmethylated" in adult as become "methylated" in adult. This is somewhat contrary to expectations that differentiation into adult tissues would reflect promoter methylation and silencing of genes not associated with the final gene expression pattern. In contrast, it suggests that the final gene expression pattern depends on extensive demethylation events during differentiation. Although methylation of gene promoter regions is associated with gene repression, gene body methylation is associated with gene expression [37,38,48]. In addition, recent reports indicate that there are extensive allelic differences in gene expression in human that result from allelic differences in methylation, due to imprinting or DNA sequence variation [49,50]. Thus, demethylation of previously methylated sites could reflect either increased or decreased gene expression, depending on site location. Our observations that demethylation is a common feature of tissue differentiation are consistent with the recent report that liver development in human is characterized predominantly by demethylation [20].

### Methylation of ES cells

ES cells are pluripotent and are derived from the blastomeres of the early embryo that are thought to be extensively demethylated due to active and passive demethylation that follows fertilization. Therefore, we decided to compare the methylation peaks from ES cells with those found in E15 embryonic tissues (brain, heart, liver, and testis) to determine the extent of methylation differences during early stages of tissue differentiation (Additional file 1). We found that the number of E15 T-DMRs (1,769) is less than half of that found in adult (4,686), which is consistent with E15 tissues being generally less methylated than adult. ES cells also had a low level of methylation with 981 T-DMRs. However, somewhat surprisingly, ES cells had more methylation peaks (981, Additional file 1) than any of the E15 tissues. This would suggest that many genomic sites that are methylated in ES cells become demethylated during early development. We previously found that many (60%) of the adult T-DMRs identified by RLGS were methylated in ES cells, suggesting these were demethylated during tissue differentiation [32]. It is also possible that some or many of the sites that are methylated in ES cells are due to growth in tissue culture. It has previously been shown that tumor cells grown in tissue culture accumulate excessive aberrant methylation that is unrelated to tumorigenesis [40]. Also, growth of mouse neural progenitor cells in culture after extended passages results in aberrant methylation [26] and investigators found extensive differences in the genomic methylation patterns of independently isolated human ES cell lines [20]. At the present time, it is unclear whether sites of methylation in ES cells is aberrant due to extended growth in culture or whether demethylation during early differentiation in ES cells is an important process as in later stages of tissue differentiation noted above.

Methylation analysis of stem cells revealed extensive Cytosine methylation in a non-CpG context [35,48,51] that was mainly located within gene bodies [35]. The non-CpG methylation appears to be mostly confined to stem cells and disappears upon differentiation. Our analysis of mouse ES cells indicated a very low level of intragenic CpGi methylation. This suggests that most of the non-CpG gene body methylation was in non-CpGi intragenic regions that were not on the NimbleGen array or that our analysis did not resolve non-CpG methylation.

### Non-Promoter and non-CpGi methylation in the Hoxa gene cluster

A 100 Kb region on chromosome 6 that includes the *Hoxa *gene cluster is essentially a tiling array on the NimbleGen promoter plus CpGi array. This region includes 16 RefSeq genes and 22 UCSC-annotated CpGi regions (Additional file 6). Somewhat surprisingly all 8 methylation peaks were in non-CpGi regions and only one was in a promoter region (Additional file 6 Additional file 7). In addition, developmental analysis of *Hoxa *gene methylation indicated stage-specific methylation differences **(**Figure 3). These differentially methylated regions include 3' exons and intron regions. Since these regions are highly conserved and *Hox *genes are known to have important roles in development [41,42], these results suggest that methylation in non-promoter, non-CpGi regions may have novel, currently undefined roles in regulating development. A recent report also indicates differential development-associated methylation within *Hox *gene clusters in human [48].

### DNA methylation and alternative promoter use

Interrogation of the MeDIP/NimbleGen array data suggests that DNA methylation may be associated with alternative promoter use. Analysis of methylation within the Protocadherin gene clusters on chromosome 18 indicates extensive methylation within CpGi promoter and non-CpGi promoter regions, particularly within somatic tissues (Additional file 8 Additional file 9). Some differential tissue specific methylation in this region is also noted. Pcdha mRNAs are synthesized by the activation of one of the alternative promoters on only one of the two chromosomes resulting in monoallelic expression [52]. Although the mechanistic basis for this selection is unknown, it is hypothesized that it provides a foundation for neuron adhesive diversity that is required for complex synaptic interactions [52]. Recent genome-wide methylation analysis using CHARM [25] also indicates an association between tissue specific DNA methylation and alternative transcripts. That study indicated that most tissue specific differentially methylated regions were located in CpGi shores and that 68% of the shores were within 500 bp of alternative promoter sites.

### The impact of T-DMRs and DS-DMRs on gene expression

To understand the biological function of T-DMRs and DS-DMRs, one obvious approach is to examine their impact on gene expression. Efforts to address this question at a genome scale is complicated by several factors that can obscure correlations.

First, one gene may be subjected to DNA methylation in multiple regions with the T-DMR or DS-DMR in question being just one of those. Therefore, the level of gene expression may depend on methylation status in other regions of the gene. Second, existence of multiple splicing isoforms, particularly those associated with alternative promoters, as well as the use of multiple expression probes for the same gene makes this one-gene vs. one DMR association analysis very challenging. Last but not least, DNA methylation is not the only factor affecting the gene expression. In other words, the lack of DNA methylation in one of the promoter region does not necessarily confer gene activation, as the expression can be limited by other factors, for instance the lack of the required transcriptional factor(s). These complications may be responsible for the highly diverse situations we observed between the occurrence of T-DMRs/DS-DMRs and the expression level of their associated genes on a gene-by-gene basis. Despite these complications, our preliminary analysis does seem to reveal a few novel insights. First, it appears that T-DMRs are associated with lower gene expression in non-CpGi promoter regions. Second, there may be some differences between T-DMRs and DS-DMRs from earlier developmental stages in the manner they impact gene expression. Certainly, extensive data analysis using more data sets representing more tissues, as well as experimental validation is needed to confirm these observations.

## Conclusions

Overall, our results indicate that there are extensive differences in DNA methylation between adult tissues and within tissues during development. These results indicate that differential DNA methylation is a dynamic process involving both methylation and demethylation events. The prominence of demethylation in adult tissues of genomic regions that are methylated at earlier stages of development was particularly striking. Even though proliferative changes in cell populations may underlie many apparent methylation differences during development, the results still indicate that extensive differences in methylation status are associated with development. Although the majority of the methylation differences were associated with non-CpGi promoter regions, there were also many methylation differences associated with non-promoter regions that may have novel roles in regulating development.

## Methods

### Growth of ES cells and collection of tissues, DNA and RNA preparations

ES cells were grown as previously described [32,53]. C57 BL/6J mice were purchased from Jackson Laboratory and maintained less than three generations in the department of Laboratory Animal Research facility at Roswell Park Cancer Institute under an approved IACUC protocol. Conventional pair breeding was performed, the plug was confirmed and then the male mouse was removed from the breeding cage after one day. Pregnant mice were sacrificed for embryo collection 15 days later (E15). Embryonic sacs were kept in PBS solution on ice. After the quick confirmation of development stage, embryos were subjected to dissection under the microscope (Leica MZ-125). Brain, heart, liver and testis tissues were carefully obtained from male embryos (verified later, see following). Male new born (NB) mice were collected within 24 hours after delivery and used for collection of the same tissues. 12 weeks old adult male mice (AD) were also used for adult tissue collection as described before [32]. Tissues and cells were immediately snap-frozen in liquid nitrogen and stored at - 80 °C until use.

The sex of embryonic mice was determined by using Y chromosome marker and the Zfxy genotyping. PCR was performed to confirm sex by using primers (Zfxya-': AGCTGTTTCATAGTCACAGAACTTAC, Zfy-'-11: CGAATGTGATGACTGTAGGAAGAATC, and Zfx-5'-2: AGAAAGCCATAGAATGCGATGAGTGC). PCR results showing multiple bands indicated male mice, which were then used in this study. Mixtures of 6 male mouse tissues were used for DNA isolation for E15 and NB analysis since it was not feasible to obtain enough DNA from a single E15 or NB animal. Adult tissues were collected from two separate animals. DNA was then prepared from 2 separate mixtures (E15 and NB) and 2 separate adults. Genomic DNA from all tissue samples was extracted using the DNeasy Kit (QIAGEN) according to Qiagen protocol.

### Sequenom MassARRAY quantitative methylation analysis

Sequenom MassARRAY quantitative methylation analysis was performed as previously described [32] using protocols provided by Sequenom (see Additional file 13 for a list of primers used). All oligonucleotides were purchased from Integrated DNA Technologies (Coralville, IA). Amplification of 1 μl bisulfite-treated DNA (~20 ng/ml) was performed using HotStar Taq Polymerase (Qiagen) in a 5 μl reaction volume using PCR primers at a 200 nM final concentration. PCR amplification was performed with the following parameters: 94 °C for 15 min hot start, followed by denaturing at 94 °C for 20 s, annealing at 56 °C for 30 s, extension at 72 °C for 1 min for 45 cycles, and final incubation at 72 °C for 3 min. PCR products were processed for MassARRAY analysis according to the manufacturer's instructions (Sequenom hMC) by the Microarray and Genomics Core Facility at Roswell Park Cancer Institute. In the mass spectrum, the relative amount of methylation can be calculated by comparing the signal intensity between mass signals of methylated and non-methylated template DNA. Each spot is ionized 50× per second at five different rasters with all resultant methylation calls performed by the EpiTyper software v1.0 (Sequenom). All data is transferred and stored in an Oracle database for tabulation. A minimum of two independently derived tissue DNAs were analyzed.

### Methylated DNA Immunoprecipitation analysis: MeDIP [33]

High-quality genomic DNA isolated from E15, NB and AD mouse tissues, was digested with *MseI *(TTAA) (New England Biolabs) to produce small fragments (200 - 1,000 bp) while keeping CpGi regions intact. Fragmented DNA was heat denatured to produce single-stranded DNA, and a portion of the denatured DNA stored as control (input) DNA. Monoclonal mouse anti 5-methyl cytidine ( Eurogentec) was used to immunoprecipitate methylated DNA fragments. The immune complexes were captured with Protein A Agarose beads (Invitrogen). Complexes were washed to remove nonspecifically bound material. Following elution of bound complexes, ethanol precipitation, and resuspension of MeDIP DNA, a small aliquot of DNA and control input DNA were used to amplify a known methylated DNA region and a known unmethylated DNA region by real-time PCR. The primers of Pst4, Pst6 and Pst21 that were identified by RLGS previously [32] were used in enrichment measurements according to the manufacturer's instructions (NimbelGen). Ratios between the MeDIP and input values are defined and normalized against a known unmethylated control sequence. After validating the enrichment of MeDIP DNA, MeDIP DNA and control input DNA were amplified by whole-genome amplification kit (Sigma Aldrich), followed by purification (QIAGEN Quick PCR Purification Kit) and then sent to NimbleGen for Microarray hybridization according to their standard protocol for the array.

### Data analysis

#### Raw data processing and methylation peak identification

The first stage of raw data processing was performed by NimbleGen as part of its commercial service (Detailed description is available at http://www.nimblegen.com). In brief, it involves the following 4 steps. 1) Signal extraction: Signal intensity data is extracted from the scanned images of each array using NimbleScan. 2) Calculation of log2 ratio: For each feature on the array, a corresponding scaled log_2_-ratio representing the ratio of the input signals for the experimental and test samples that were co-hybridized to the array is computed and scaled to center the ratio data around zero. Scaling is performed by subtracting the bi-weight mean for the log_2_-ratio values for all features on the array from each log_2_-ratio value. 3) Calculation of P score: From the scaled log_2_-ratio data, a fixed-length window (750 bp) is placed around each consecutive probe and the one-sided Kolmogorov-Smirnov (KS) test is applied to determine whether the probes are drawn from a significantly more positive distribution of intensity log-ratios than those in the rest of the array. The resulting score for each probe, called P score, is the -log_10 _p-value from the windowed KS test around that probe. 4) Identification of methylation peaks: This is performed by searching for regions containing at least 2 consecutive probes with a P score ≥ 2. Peaks within 500 bp of each other are merged. The processed data (P Scores) and raw data (log2 ratios) for all 24 involved samples have been deposited in GEO at NCBI under accession# GSE21415.

#### Identification of methylation peaks shared by or unique to a given set of samples

This part was mostly done using an in-house bioinformatics tool based on the following criteria and definition: 1) A methylation peak of a sample is the methylated region shared between the two biological replicates of the same sample. This requirement applies to all categories of methylation peaks; 2) A methylation peak unique to a sample or a group of samples is a methylated region that only appeared in the sample or group. It was further examined to ensure that this region does not overlap with any raw peaks (those of individual replicates from NimbleScan) of other individual samples; 3) A peak shared by one or more but not all samples in a group; 4) A methylation peak common to a group of samples. In identifying these methylation peaks, it is often necessary to break the larger overlapping raw peaks into several sub-peaks such that one part of an original peak may be identified as a common peak and another part as a unique peak.

#### Defining the location category of DMRs

To define the location of a methylation peak in context of a gene, we used the annotation data of promoters and CpGi regions of mouse genome provided by NimbleGen, which is in turn based on UCSC February 2006 (mm8) genome freeze for annotation of CpGi regions and mRNAs and their genome coordinates. Using an in-house PERL script, each peak is assigned into one of the following categories: 1) CpGi promoter, located within 500 bp distance to a CpGi and is within an annotated promoter (1 kb flanking each side of the transcription start site or TSS); 2) non-CpGi promoter, located within a promoter without CpGi or more than 500 bp away from any CpGi in the promoter; 3) Intragenic CpGi, located within 500 bp of a CpGi that locates in a gene (excluding the promoter as defined above); 4) Intergenic CpGi, located within 500 bp from a CpGi that is more than 2 kb from any annotated genes. The assignment is done in the above order of the four categories such that a given DMR belongs to only one category with the CpGi promoter having a higher priority over the rest of the categories and so on. For DMRs in tiling regions, two additional categories were added as non-CpGi intergenic and non-CpGi intragenic. DMRs in tiling regions were identified by collecting all DMRs located within the tiling regions, which include Chr6: 51,984,676-52,408,211, Chr7: 142,162,309-143,552,631 and Chr17: 12,525,776-12,614,350.

#### Gene ontology analysis and other statistical analyses

For DMRs associated with a gene (including CpGi promoter, non-CpGi promoter, and Intragenic CpGi), we examined whether a statistically significant enrichment of gene ontology - a given gene list can be observed using the DAVID tool [54]. A significant enrichment requires a Benjamini adjusted P value no more than 0.05. Scatter plots were generated using R http://www.r-project.org/ and Heat maps of P scores were generated using MeV http://www.tm4.org/mev/[55].

### Assessment of the MeDIP data quality and validation of MeDIP methylation peaks

To evaluate the overall quality of the MeDIP data, we plotted the log2 ratios (methylation signals between the IP DNA and the input DNA) and P score of all probes between biological replicates representing different samples of the same tissues from different animals. The scatter plots of log 2 ratios as shown in Additional file 14 indicates that a good level of consistency was seen among biological replicates for all examined tissues (Additional file 4), considering these represented both biological and technical replicates (Pearson Coefficient R = 0.68 to 0.88 with over half being > 0.8). The Pearson Coefficient between replicates is improved in all cases, ranging from 0.75 to 0.94, when P score was used (Additional file 4), on which the peak call was based, indicating some sporadic noise was removed by considering the signal level of neighboring probes in a larger window.

We previously identified T-DMRs using Restriction Landmark Genomic Scanning (RLGS) [19,32], and some of these RLGS T-DMRs were used as internal controls for the quality of IP DNA prior to the array hybridization. Quantitative PCR analysis of input and IP DNA samples indicated that the IP DNA from the appropriate tissue was enriched for DNA sequences previously shown to have tissue specific DNA methylation (presence or absence of methylated DNA fragments at Pst6 and Pst21 were confirmed prior to the hybridization; data not shown). Additional file 15 shows the results of MeDIP methylation analysis in the regions of four previously identified RLGS T-DMRs (Pst4, Pst10, Pvu4 and Pvu5) that were confirmed by Sequenom MassArray quantitative methylation analysis [32]. These results are consistent with both RLGS analysis and the Sequenom analysis of bisulfite treated DNA [32]. Among the 68 RLGS-TDMRs, 38 overlap with the probes on the array (allowing a distance of 750 bp the window size we used for peak identification), of which 27 overlap with the MeDIP DS-DMRs or T-DMRs. Among the 11 not picked up by MeDIP, 5 are in the repetitive regions and would not be included on the array. Therefore, the sensitivity level of our MeDIP in reference to RLGS is 27/33 or 82%.

Sequenom MassArray quantitative methylation analysis of bisulfite treated DNA was performed as an additional means to confirm DNA methylation peaks obtained by MeDIP/NimbleGen Array for both selected and randomly chosen MeDIP methylated regions. The Sequenom MassArray results were consistent with the MeDIP results in 19 of 23 peaks that were evaluated (18 randomly chosen and 5 selected peaks). An example of the Sequenom MassArray results for a CpGi promoter region is shown in Figure 1, and for a randomly selected non-CpGi promoter region (Additional file 16) indicating the two methods are in agreement.

We also interrogated MeDIP data within imprinted regions that are known to be methylated in somatic tissues. Almost the entire imprinted region on Chr 7 that includes the *Kcnq1 *gene is represented in a tiling array (1,390 kb) on the NimbleGen promoter + CpGi array. A 40 kb portion of the *Kcnq1 *that contains KvDMR is shown in Additional file 2. The KvDMR is an imprinted control region of approximately 2 Kb (boxed region) that is maternally methylated and has previously been shown to be unmethylated in sperm [56]. Consistent with these earlier results, the MeDIP/NimbleGen array results clearly indicate that DNA from the testis is not methylated in this region. Similarly, the imprinting control region associated with *Igf2r *is also unmethylated in testis [57] (Additional file 17). Since only the maternal allele is methylated in these imprinted regions, MeDIP is capable of detecting methylation in a single allele.

### Analysis of relationship between T-DMRs/DS-DMRs and gene expression

Two gene expression data sets, GSE9954 and GSE13149, that are deemed suitable for our purpose were identified and retrieved from the NCBI GEO database. For GSE9954, we used the normalized expression values provided in GEO by the authors and performed the distribution pattern and statistical analysis of expression for genes associated with all T-DMRs from each tissue. Specifically, for T-DMRs from each of the 3 location categories, i.e., the CpGi promoter, the non-CpGi promoter and intra-genic CpGi, in a tissue, we compared the expression level and distribution pattern of their associated genes in that tissue with that of the same set of genes in three other tissues, as well as gene associated T-DMRs in other two location categories, based on box plot and t-test using the R package. For GSE13149, the raw Cel files were downloaded from GEO for samples at E15.5, Day0 and adult mice, which presumably correspond to our E15, new born (NB) and adult samples, and were processed for gcRMA and quantile normalization using the BioConductor R page. The expression data in association with liver DS-DMRs was analyzed in a manner similar to T-DMRs.

## Authors' contributions

PL: performed most of the bioinformatics data analysis and interpretation and contributed in writing the manuscript; FS: performed most of the molecular analysis including preparation for MeDIP and MassARRAY analysis and contributed in writing, SG: performed validation experiments, EM: performed data analysis; MQ: developed the computational tools for identifying methylation peaks shared by or unique to different sample groups, SM: performed ES cell and DNA preparation and validation experiments, KF and IJ: performed tissue preparation, HN: performed supervision of most of molecular biological analysis and contributed in writing the manuscript, WH: performed most of writing and supervision of both bioinformatics and molecular analysis. All authors read and approved the final manuscript.

## Supplementary Material

Additional file 1 **E15 and ES T-DMRs**. Click here for file

Additional file 2 **MeDIP/NimbleGen Promoter + CpGi Array (Tiling region): Methylation analysis of KvDMR region**. The scaled log2 ratio of the 40 Kb region on chromosome 7 near KvDMR is shown. The numbers on the top indicate the genomic position. The rectangle indicates the position of the KvDMR that includes two CpGi regions. Two independent tissues were taken from different mice. Previous studies indicate that the KvDMR is methylated in somatic tissue and unmethylated in sperm [57]. Click here for file

Additional file 3 **T-DMRs in tiling regions**. Click here for file

Additional file 4 **Pearson Coefficient and common peak number between replicates**. Click here for file

Additional file 5 **a: Overlaps between different tissues for DS-DMRs with testis excluded; b:Overlaps between tissues for DMRs shared by all developmental stages in a tissue**. Click here for file

Additional file 6 **T-DMRs within the Hoxa gene cluster**. The MeDIP methylation profile for the *Hoxa *gene cluster within the 52.08 to 52.20 Mb region on chromosome 6 is shown. The locations of CpGi regions, *Hoxa *gene transcripts from a1 to a13 and transcription direction are indicated. The numbers on the bottom from 1 to 8 indicate the position of methylation peaks (represented by the log2 ratio) corresponding to those listed in Additional file 7. Click here for file

Additional file 7 **Methylation peaks locations in the Hoxa gene cluster**. Click here for file

Additional file 8 **DNA methylation and alternative promoters for *Pcdha *genes**. Methylation profile for *Pcdha *genes is shown in a similar manner as in Additional file 2. See also Additional file 9. Click here for file

Additional file 9 **Methylation peaks locations in the Protocadherin gene cluster**. Click here for file

Additional file 10 **Relationship between T-DMRs and expression of their associated genes**. The expression of the genes associated with liver unique T-DMRs were compared to that of the same gene set in the three other tissues broken down into the CpGi promoter (CpGi_P), non-CpGi promoter (NCpGi_P) and intra-genic CpGi (Intra_CpGi) groups using boxplots (panel A, B and C, respectively). Comparison was also made for T-DMRs associated genes across different location groups within liver (Panel D). The p values of pairwise t-tests between liver and each of the other tissue (panels A-C) and between each pair of location groups (panel C) are provided on the right side of the boxplots with those no great than 0.05 shown in bold font. "NDMR" in panel C refers to genes associated with all genes not associated with liver unique T-DMRs. Click here for file

Additional file 11 **Gene ontology for T-DMRs in adult**. Click here for file

Additional file 12 **Gene ontology for DS-DMRs subjected to methylation or demethylation in adult**. Click here for file

Additional file 13 **A list of Sequenom MassARRAY primers used for Validation**. Click here for file

Additional file 14 **Scatter plots of log2 ratios of two replicates for brain tissues at E15, NB, and AD stages**. Log2 ratio of all probes for the two replicate samples of brain at all stages were plotted. In each plot, data points in red rectangular area are those showing log2 ratio ≥ 1 (i.e. ratio ≥2) in both samples. Pearson Coefficient and the common methylation peak number are provided on the top of each plot. Click here for file

Additional file 15 **MeDIP/NimbleGen Promoter + CpGi Array: Methylation analysis of RLGS T-DMR loci**. The MeDIP methylation profile including scaled log2 ratio and methylation peaks of four RLGS T-DMR loci are shown (Pst4, Pst10, Pvu4 and Pvu5). The loci were identified as T-DMRs by RLGS [32]. The CpG island, genomic location, relevant gene and the direction of each transcript are indicated. The log2 ratio is the ratio of signals for the input and immunoprecipitated DNA test samples that were co-hybridized to the array. RLGS T-DMRs were also confirmed by Sequenom MassARRAY ([32], and data not shown). Click here for file

Additional file 16 **Comparison of methylation data between MeDIP/NimbleGen Promoter + CpGi Array and Sequenom MassARRAY: Non CpGi promoter region (randomly selected)**. Data similar to that presented in Figure 1 for a randomly selected locus, the non-CpGi promoter region of Gm1070 that is methylated in liver and ES, but not in testis. Click here for file

Additional file 17 **MeDIP/NimbleGen Promoter + CpGi Array (Tiling region): Methylation analysis of Igf2r imprint control region**. The MeDIP methylation profile of a region on chromosome 17 that includes Igf2r and BC009123 is shown. The numbers on the top indicate the genomic position. The CpGi in the rectangle is located close to the promoter region of BC009123 and the second intron of *Igf2r*. Methylation within the CpGi is associated with repression of transcription of *Airn *antisense RNA located somewhat downstream of TSS for BC009123. The imprinting control region (rectangle) is monoallelically methylated in somatic tissue and unmethylated in testis [57]. Click here for file
