# Supplementary material for: Genome-wide survey reveals dynamic widespread tissue-specific changes in DNA methylation during development
Source: BMC Genomics. 2011 May 11;12:231. doi: 10.1186/1471-2164-12-231 (PMC3118215; doi:10.1186/1471-2164-12-231)
Supplement: Additional file 2 — MeDIP/NimbleGen Promoter + CpGi Array (Tiling region): Methylation analysis of KvDMR region. The scaled log2 ratio of the 40 Kb region on chromosome 7 near KvDMR is shown. The numbers on the top indicate the genomic position. The rectangle indicates the position of the KvDMR that includes two CpGi regions. Two independent tissues were taken from different mice. Previous studies indicate that the KvDMR is methylated in somatic tissue and unmethylated in sperm [57]. [file 1471-2164-12-231-S2.PPT]

## Slide 1
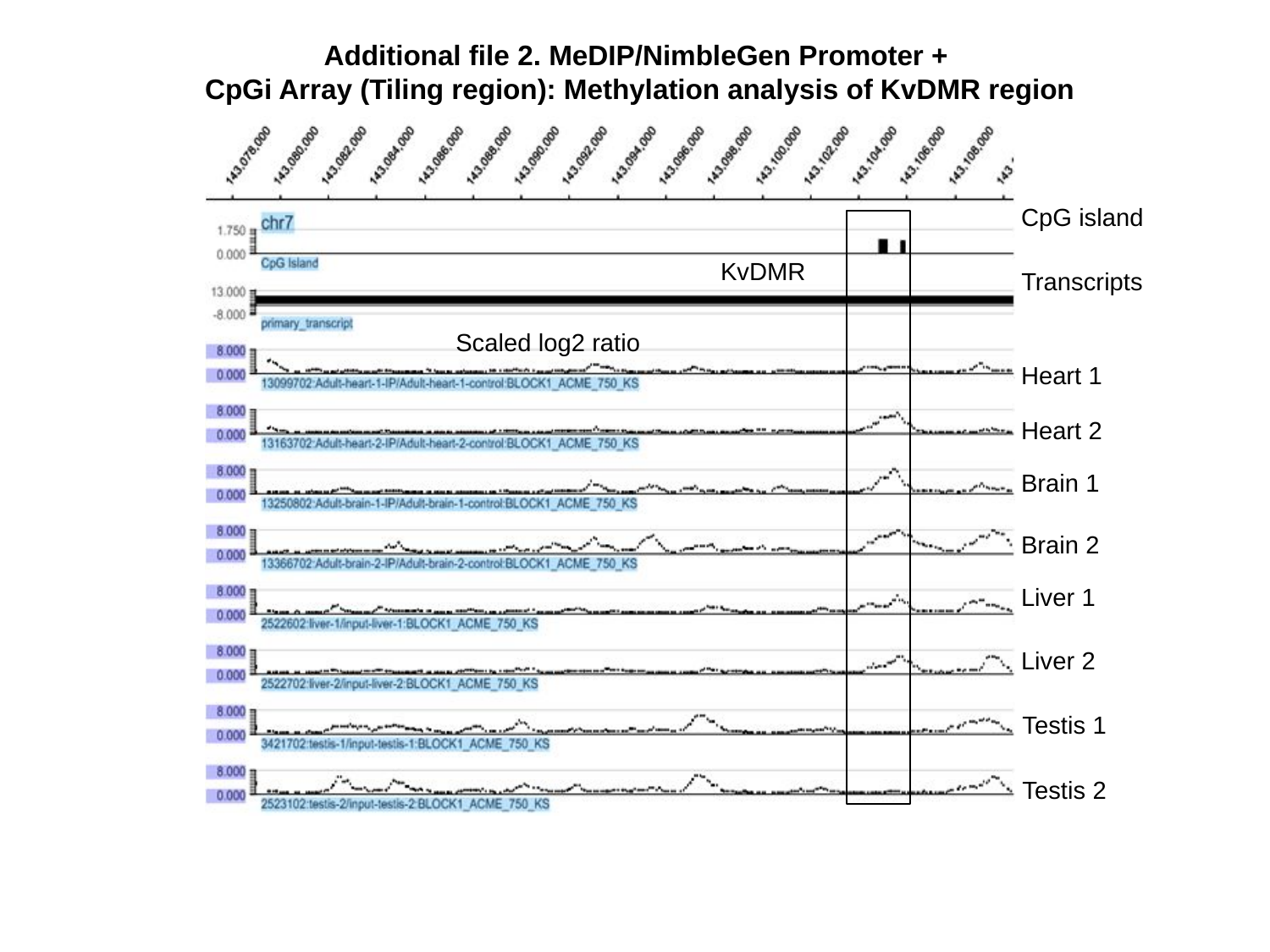

Additional file 2. MeDIP/NimbleGen Promoter +
CpGi Array (Tiling region): Methylation analysis of KvDMR region
CpG island
KvDMR
Transcripts
Scaled log2 ratio
Heart 1
Heart 2
Brain 1
Brain 2
Liver 1
Liver 2
Testis 1
Testis 2
