# Supplementary material for: Genome-wide survey reveals dynamic widespread tissue-specific changes in DNA methylation during development
Source: BMC Genomics. 2011 May 11;12:231. doi: 10.1186/1471-2164-12-231 (PMC3118215; doi:10.1186/1471-2164-12-231)
Supplement: Additional file 6 — T-DMRs within the Hoxa gene cluster. The MeDIP methylation profile for the Hoxa gene cluster within the 52.08 to 52.20 Mb region on chromosome 6 is shown. The locations of CpGi regions, Hoxa gene transcripts from a1 to a13 and transcription direction are indicated. The numbers on the bottom from 1 to 8 indicate the position of methylation peaks (represented by the log2 ratio) corresponding to those listed in Additional file 7. [file 1471-2164-12-231-S6.PPT]

## Slide 1
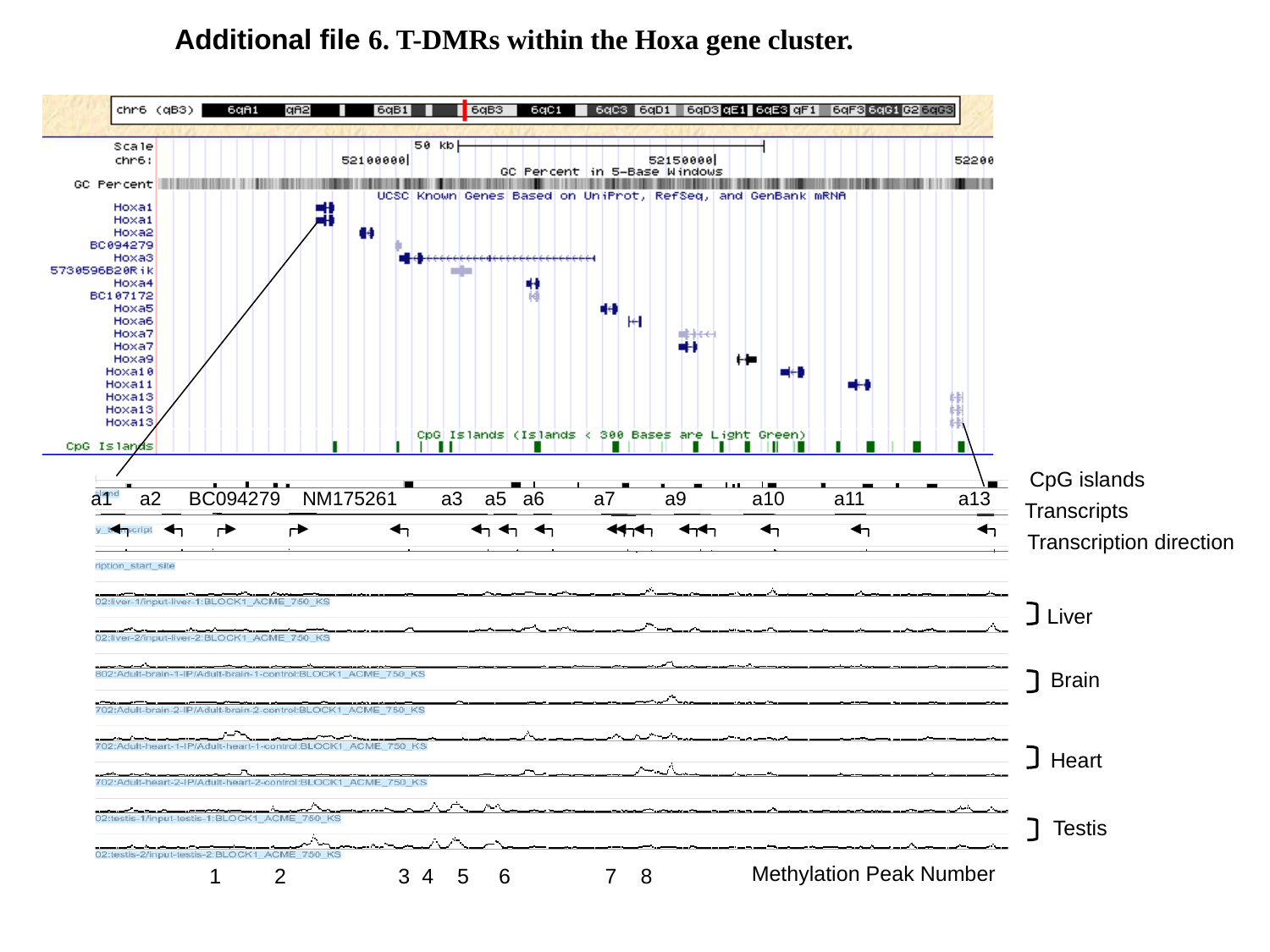

Additional file 6. T-DMRs within the Hoxa gene cluster.
CpG islands
a1 a2 BC094279 NM175261 a3 a5 a6 a7 a9 a10 a11 a13
Transcripts
Transcription direction
Liver
Brain
Heart
Testis
Methylation Peak Number
1 2 3 4 5 6 7 8
