# Supplementary material for: Genome-wide survey reveals dynamic widespread tissue-specific changes in DNA methylation during development
Source: BMC Genomics. 2011 May 11;12:231. doi: 10.1186/1471-2164-12-231 (PMC3118215; doi:10.1186/1471-2164-12-231)
Supplement: Additional file 8 — DNA methylation and alternative promoters for Pcdha genes. Methylation profile for Pcdha genes is shown in a similar manner as in Additional file 2. See also Additional file 9. [file 1471-2164-12-231-S8.PPT]

## Slide 1
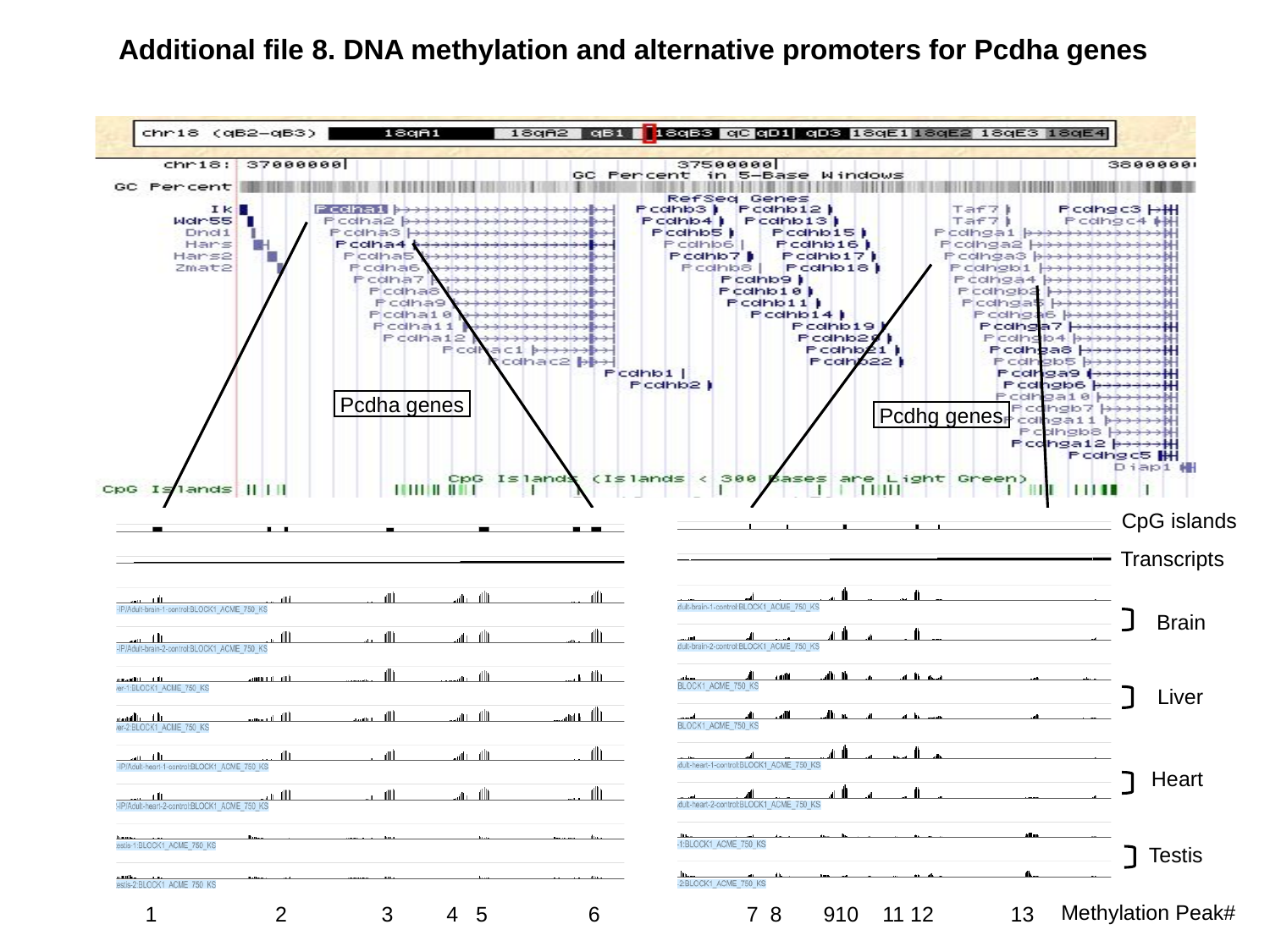

Additional file 8. DNA methylation and alternative promoters for Pcdha genes
Pcdha genes
Pcdhg genes
CpG islands
Transcripts
Brain
Liver
Heart
Testis
Methylation Peak#
 1 2 3 4 5 6
 7 8 910 11 12 13
