# Supplementary material for: Genome-wide survey reveals dynamic widespread tissue-specific changes in DNA methylation during development
Source: BMC Genomics. 2011 May 11;12:231. doi: 10.1186/1471-2164-12-231 (PMC3118215; doi:10.1186/1471-2164-12-231)
Supplement: Additional file 10 — Relationship between T-DMRs and expression of their associated genes. The expression of the genes associated with liver unique T-DMRs were compared to that of the same gene set in the three other tissues broken down into the CpGi promoter (CpGi_P), non-CpGi promoter (NCpGi_P) and intra-genic CpGi (Intra_CpGi) groups using boxplots (panel A, B and C, respectively). Comparison was also made for T-DMRs associated genes across different location groups within liver (Panel D). The p values of pairwise t-tests between liver and each of the other tissue (panels A-C) and between each pair of location groups (panel C) are provided on the right side of the boxplots with those no great than 0.05 shown in bold font. "NDMR" in panel C refers to genes associated with all genes not associated with liver unique T-DMRs. [file 1471-2164-12-231-S10.PPT]

## Slide 1
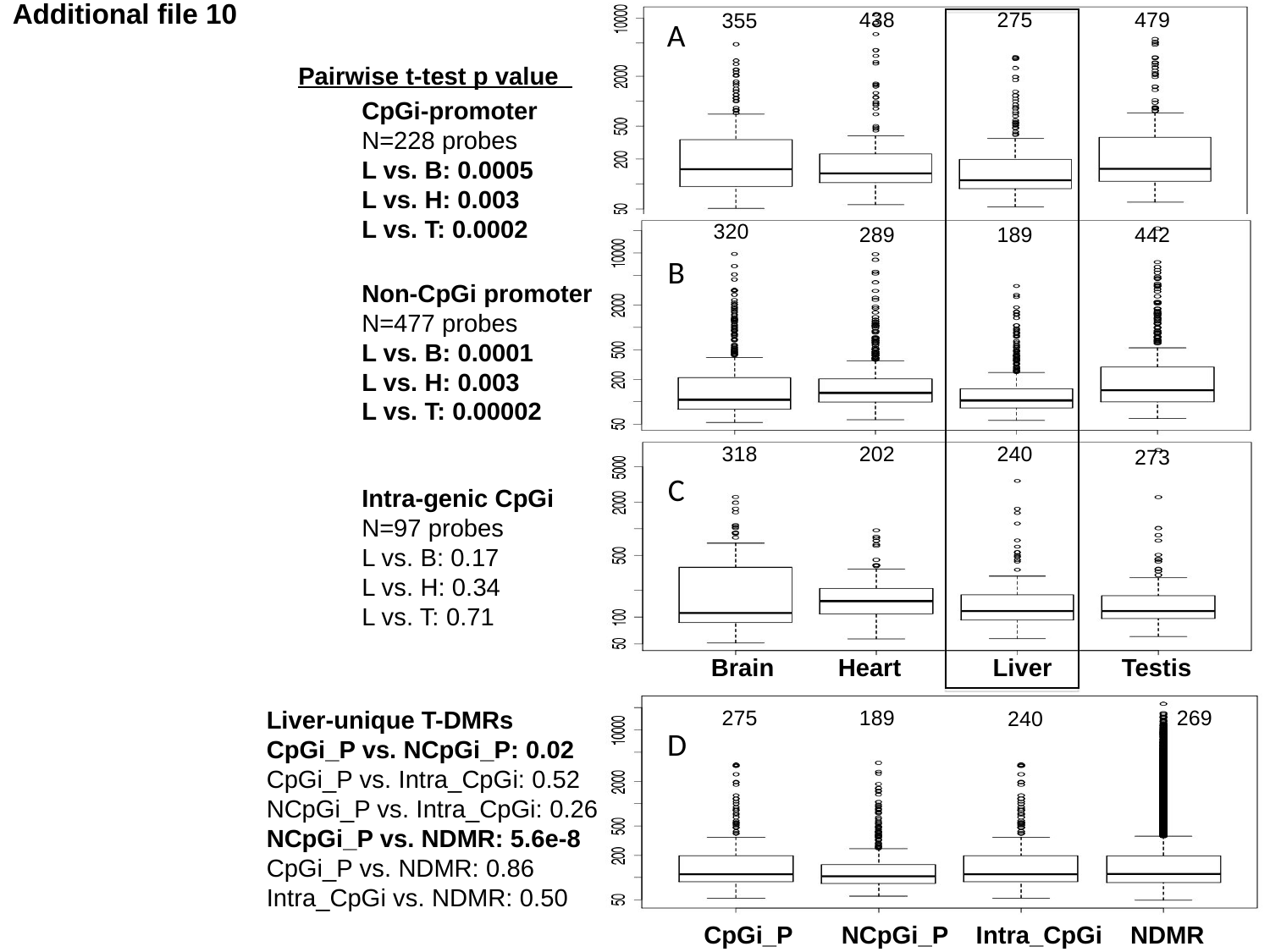

438
275
479
Additional file 10
355
A
Pairwise t-test p value
CpGi-promoter
N=228 probes
L vs. B: 0.0005
L vs. H: 0.003
L vs. T: 0.0002
320
289
189
442
B
Non-CpGi promoter
N=477 probes
L vs. B: 0.0001
L vs. H: 0.003
L vs. T: 0.00002
318
202
240
273
C
Intra-genic CpGi
N=97 probes
L vs. B: 0.17
L vs. H: 0.34
L vs. T: 0.71
Brain 	Heart 	 Liver Testis
Liver-unique T-DMRs
CpGi_P vs. NCpGi_P: 0.02
CpGi_P vs. Intra_CpGi: 0.52
NCpGi_P vs. Intra_CpGi: 0.26
NCpGi_P vs. NDMR: 5.6e-8
CpGi_P vs. NDMR: 0.86
Intra_CpGi vs. NDMR: 0.50
275
189
269
240
D
CpGi_P NCpGi_P Intra_CpGi NDMR
