# Supplementary material for: Genome-wide survey reveals dynamic widespread tissue-specific changes in DNA methylation during development
Source: BMC Genomics. 2011 May 11;12:231. doi: 10.1186/1471-2164-12-231 (PMC3118215; doi:10.1186/1471-2164-12-231)
Supplement: Additional file 14 — Scatter plots of log2 ratios of two replicates for brain tissues at E15, NB, and AD stages. Log2 ratio of all probes for the two replicate samples of brain at all stages were plotted. In each plot, data points in red rectangular area are those showing log2 ratio ≥ 1 (i.e. ratio ≥2) in both samples. Pearson Coefficient and the common methylation peak number are provided on the top of each plot. [file 1471-2164-12-231-S14.PPT]

## Slide 1
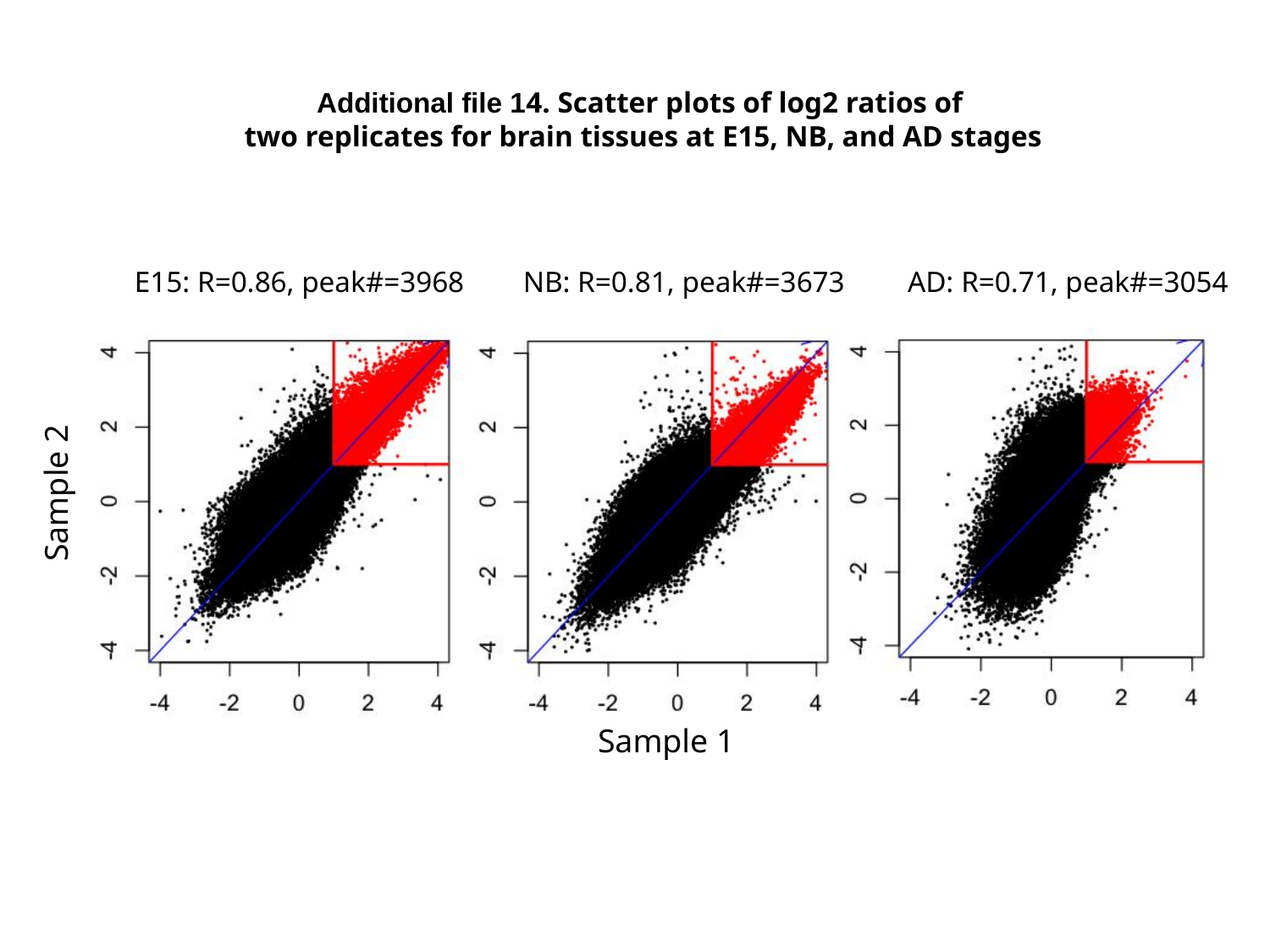

Additional file 14. Scatter plots of log2 ratios of
two replicates for brain tissues at E15, NB, and AD stages
E15: R=0.86, peak#=3968
NB: R=0.81, peak#=3673
AD: R=0.71, peak#=3054
Sample 2
Sample 1
