# Supplementary material for: Genome-wide survey reveals dynamic widespread tissue-specific changes in DNA methylation during development
Source: BMC Genomics. 2011 May 11;12:231. doi: 10.1186/1471-2164-12-231 (PMC3118215; doi:10.1186/1471-2164-12-231)
Supplement: Additional file 15 — MeDIP/NimbleGen Promoter + CpGi Array: Methylation analysis of RLGS T-DMR loci. The MeDIP methylation profile including scaled log2 ratio and methylation peaks of four RLGS T-DMR loci are shown (Pst4, Pst10, Pvu4 and Pvu5). The loci were identified as T-DMRs by RLGS [32]. The CpG island, genomic location, relevant gene and the direction of each transcript are indicated. The log2 ratio is the ratio of signals for the input and immunoprecipitated DNA test samples that were co-hybridized to the array. RLGS T-DMRs were also confirmed by Sequenom MassARRAY ([32], and data not shown). [file 1471-2164-12-231-S15.PPT]

## Slide 1
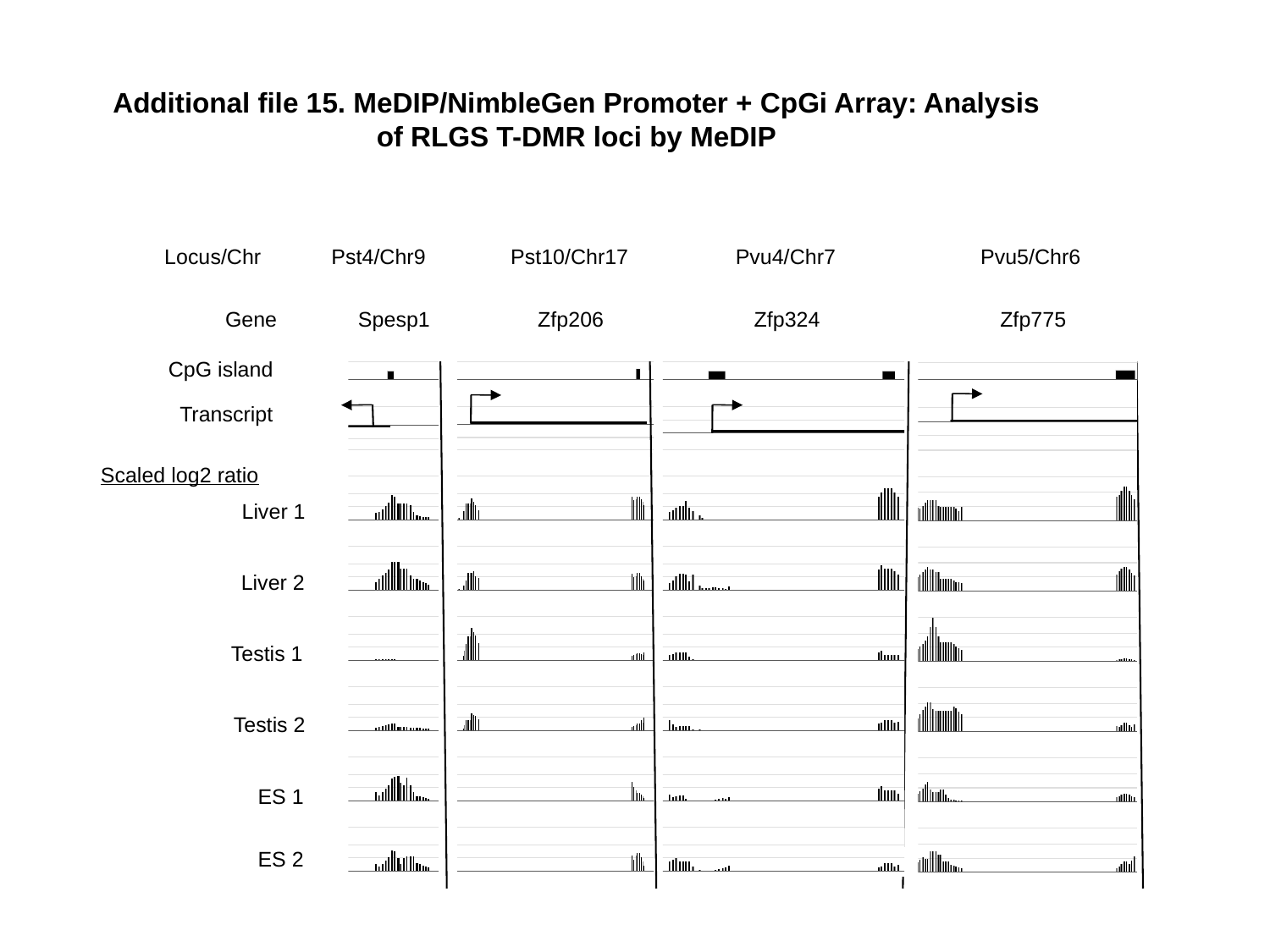

Additional file 15. MeDIP/NimbleGen Promoter + CpGi Array: Analysis of RLGS T-DMR loci by MeDIP
Locus/Chr
Pst4/Chr9
Pst10/Chr17
Pvu4/Chr7
Pvu5/Chr6
Gene
Spesp1
Zfp206
Zfp324
Zfp775
CpG island
Transcript
Scaled log2 ratio
Liver 1
Liver 2
Testis 1
Testis 2
ES 1
ES 2
