# Supplementary material for: Genome-wide survey reveals dynamic widespread tissue-specific changes in DNA methylation during development
Source: BMC Genomics. 2011 May 11;12:231. doi: 10.1186/1471-2164-12-231 (PMC3118215; doi:10.1186/1471-2164-12-231)
Supplement: Additional file 16 — Comparison of methylation data between MeDIP/NimbleGen Promoter + CpGi Array and Sequenom MassARRAY: Non CpGi promoter region (randomly selected). Data similar to that presented in Figure 1 for a randomly selected locus, the non-CpGi promoter region of Gm1070 that is methylated in liver and ES, but not in testis. [file 1471-2164-12-231-S16.PPT]

## Slide 1
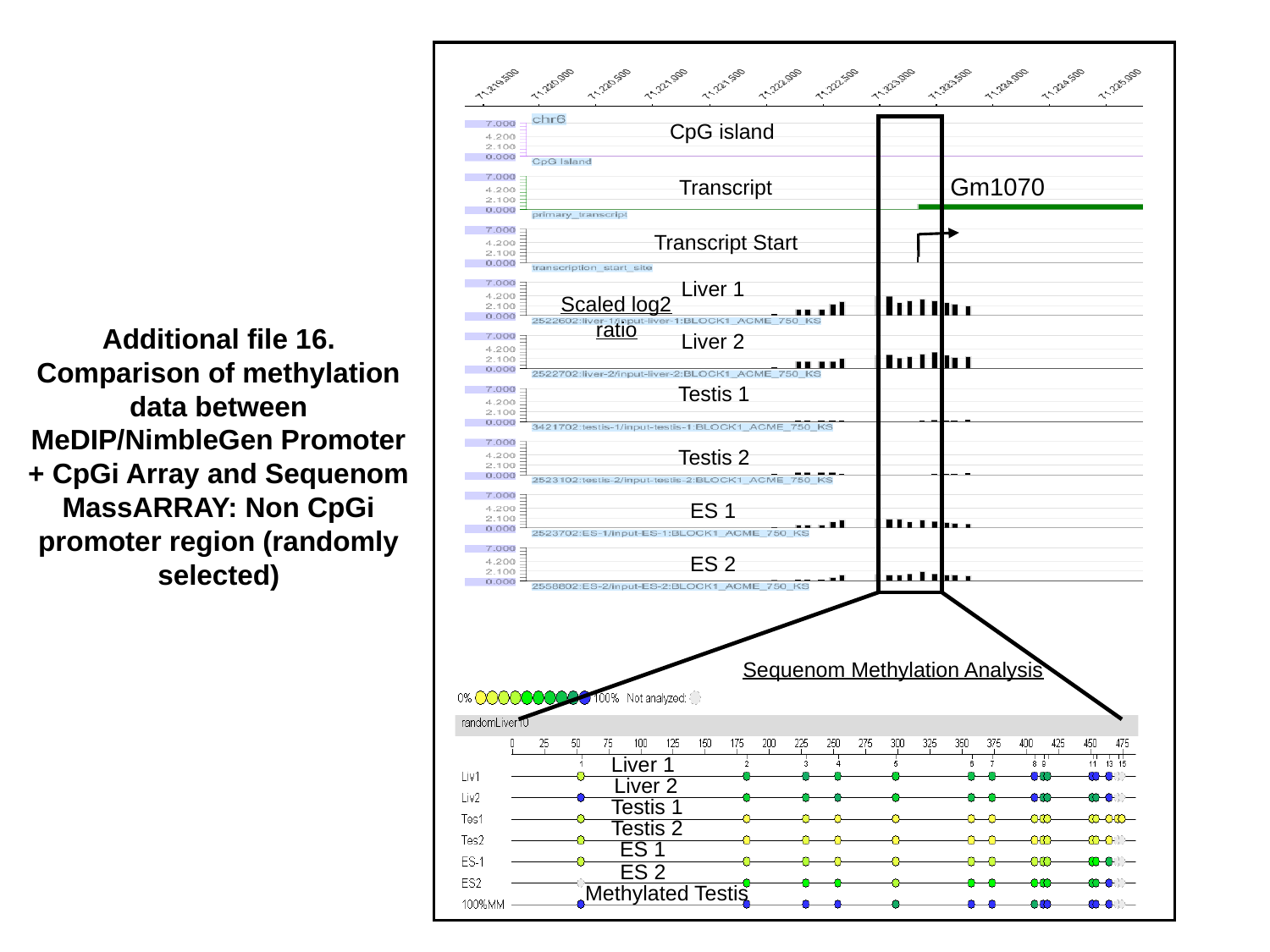

CpG island
Gm1070
Transcript
Transcript Start
Liver 1
Scaled log2 ratio
Additional file 16. Comparison of methylation data between MeDIP/NimbleGen Promoter + CpGi Array and Sequenom MassARRAY: Non CpGi promoter region (randomly selected)
Liver 2
Testis 1
Testis 2
ES 1
ES 2
Sequenom Methylation Analysis
Liver 1
Liver 2
Testis 1
Testis 2
ES 1
ES 2
Methylated Testis
