# Supplementary material for: Genome-wide survey reveals dynamic widespread tissue-specific changes in DNA methylation during development
Source: BMC Genomics. 2011 May 11;12:231. doi: 10.1186/1471-2164-12-231 (PMC3118215; doi:10.1186/1471-2164-12-231)
Supplement: Additional file 17 — MeDIP/NimbleGen Promoter + CpGi Array (Tiling region): Methylation analysis of Igf2r imprint control region. The MeDIP methylation profile of a region on chromosome 17 that includes Igf2r and BC009123 is shown. The numbers on the top indicate the genomic position. The CpGi in the rectangle is located close to the promoter region of BC009123 and the second intron of Igf2r. Methylation within the CpGi is associated with repression of transcription of Airn antisense RNA located somewhat downstream of TSS for BC009123. The imprinting control region (rectangle) is monoallelically methylated in somatic tissue and unmethylated in testis [57]. [file 1471-2164-12-231-S17.PPT]

## Slide 1
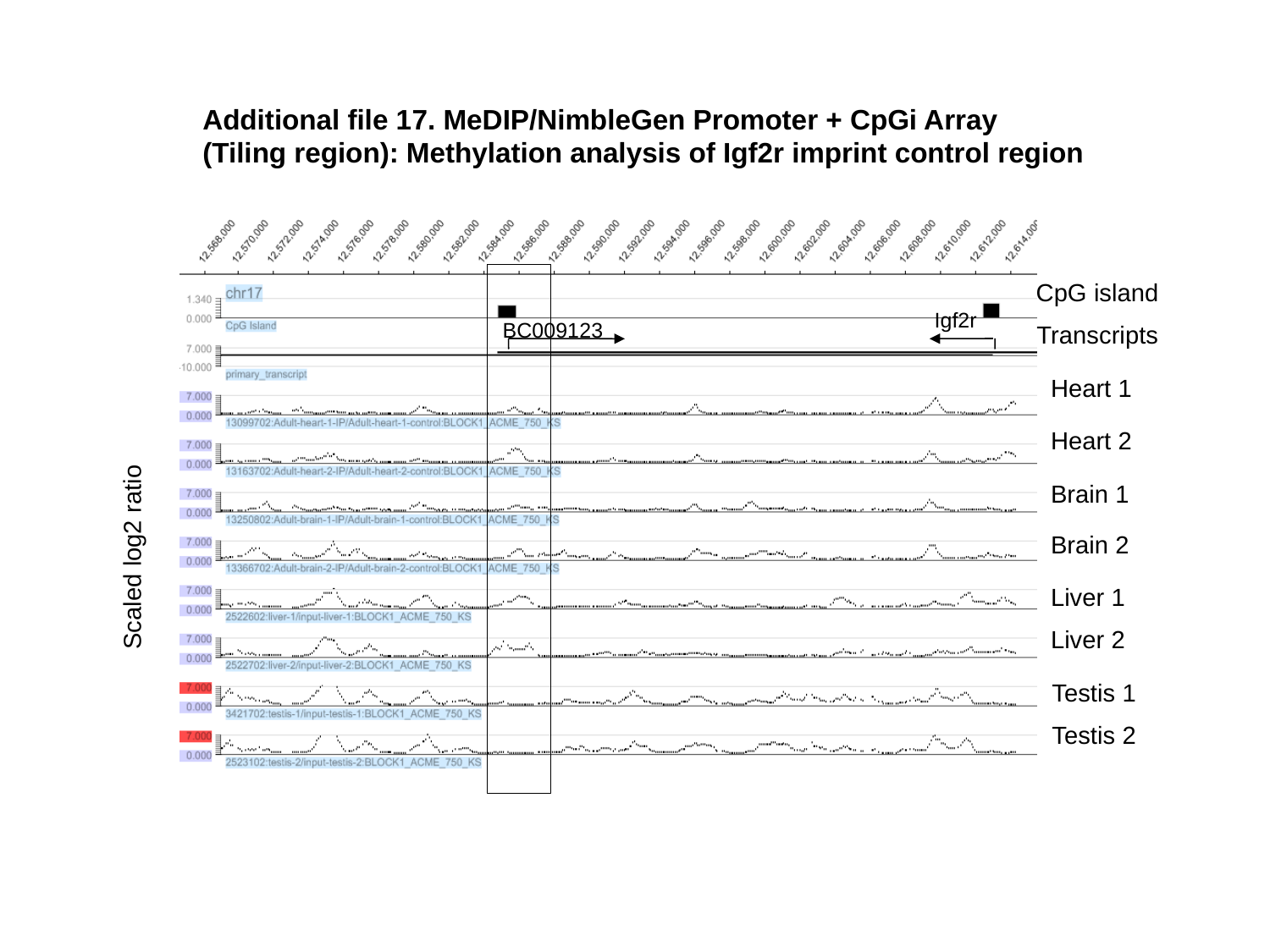

Additional file 17. MeDIP/NimbleGen Promoter + CpGi Array
(Tiling region): Methylation analysis of Igf2r imprint control region
CpG island
Igf2r
BC009123
Transcripts
Heart 1
Heart 2
Brain 1
Brain 2
Scaled log2 ratio
Liver 1
Liver 2
Testis 1
Testis 2
